# Origin of macroscopic adhesion in organic light-emitting diodes analyzed at different length scales

**DOI:** 10.1038/s41598-018-24889-9

**Published:** 2018-04-23

**Authors:** Sungho Kim, Seongjae Park, Wanheui Lee, Owoong Kwon, Shang-U. Kim, Youngtae Choi, Minyoung Yoon, Jongwoo Park, Yunseok Kim

**Affiliations:** 1 School of Advanced Materials Science and Engineering, Sungkyunkwan University (SKKU), Suwon, 16419 Republic of Korea; 2 Samsung Display Co., Ltd, Asan, 31454 Republic of Korea

## Abstract

Organic light-emitting diodes (OLEDs) have been widely studied because of their various advantages. OLEDs are multi-layered structures consisting of organic and inorganic materials arranged in a heterojunction; the nature of adhesion at their heterogeneous interfaces has a significant effect on their properties. In this study, the origin of macroscopic adhesion was explored in OLEDs using a combination of microscopy techniques applied at different length scales. The different techniques allowed the identification of layers exposed by a peel test, which aided direct characterization of their macroscopic adhesion. Further, the contribution of each exposed layer to macroscopic adhesion could be determined through an analysis of photographic images. Finally, analysis of the local roughness and adhesion confirmed that the interface between an anode and emission layer could play a predominant role in determining the nature of macroscopic adhesion in OLEDs. These results may provide guidelines for exploring the origin of macroscopic adhesion properties through a combination of various microscopy techniques.

## Introduction

Since their invention, organic light-emitting diodes (OLEDs) have been studied extensively because of their low power consumption, high brightness, and improved contrast ratio in numerous display applications such as televisions and cellular phones^1–4^. An OLED differs from a conventional liquid crystal display in various ways, including in its structure and the components used^5,6^. In particular, since OLEDs are based on organic materials, their life expectancy and performance depend on the transport of electrons and holes across their interfaces^7–10^. Thus, the heterogeneous interfacial properties between organic materials and semiconductors or metals continue to be an important factor in determining the life expectancy and performance of an OLED^11–15^. Furthermore, since an OLED can be easily bent when used as an organic material base, polymeric substrates have been investigated to potentially replace glass substrates in flexible displays^16–18^. These research trends suggest that an analysis of the adhesion properties is necessary to provide not only insight into the adhesion properties between different interfacial layers but also direction to the application of OLEDs on other materials.

Several methods are available to measure the adhesion properties of materials, including peel tests and contact angle measurements^13,19–21^. However, these conventional methods are limited to the analysis of macroscopic adhesion properties. Even though the macroscopic adhesion properties are primarily determined by weak adhesion interface, it is difficult to determine what interface types lead to weak macroscopic adhesion, much less the individual adhesion properties of each layer or interface. Therefore, a complementary approach based on microscopic analysis tools is necessary to better understand macroscopic adhesion. However, since device fabrication and operation occur at the nanoscale level and nanoscopic factors such as surface roughness and local adhesion can significantly affect macroscopic adhesion properties, the analysis of adhesion must also be performed at the nanoscale. Accordingly, atomic force microscopy (AFM) has emerged as a local approach for observing surface roughness and local adhesion. Although a few studies on the local adhesion of OLEDs have been reported^22,23^, the correlation between macroscopic adhesion and local physical properties remains unclear. Furthermore, although macroscopic adhesion needs to be studied at various length scales, as discussed above, a combination of various microscopy tools over different length scales has been rarely applied in the analysis of macroscopic adhesion of OLEDs.

In this study, we investigated the origin of local contributions to macroscopic adhesion in OLEDs by a combination of various microscopy techniques at different length scales. A peel test was performed to study macroscopic adhesion in OLEDs. Then, the exposed layers were identified using a common digital camera, optical microscopy (OM), and cross-sectional scanning electron microscope (SEM) imaging. Further, each area portion of exposed layers of the OLEDs was calculated using image analysis. The results on the calculation of the area portion showed that an exposed anode may be responsible for macroscopic adhesion. Additionally, the surface roughness and adhesion force on the exposed layers were analyzed using AFM. Through the AFM measurements, we confirmed that the exposed anode, i.e., the interface between the anode and emission layer (EL), had a predominant effect on the macroscopic adhesion properties of OLEDs. These results may provide useful information about the macroscopic adhesion of OLEDs, while providing guidelines for the exploration of the origin of macroscopic adhesion properties through a combination of various microscopy techniques.

## Results and Discussion

**Figure 1:**
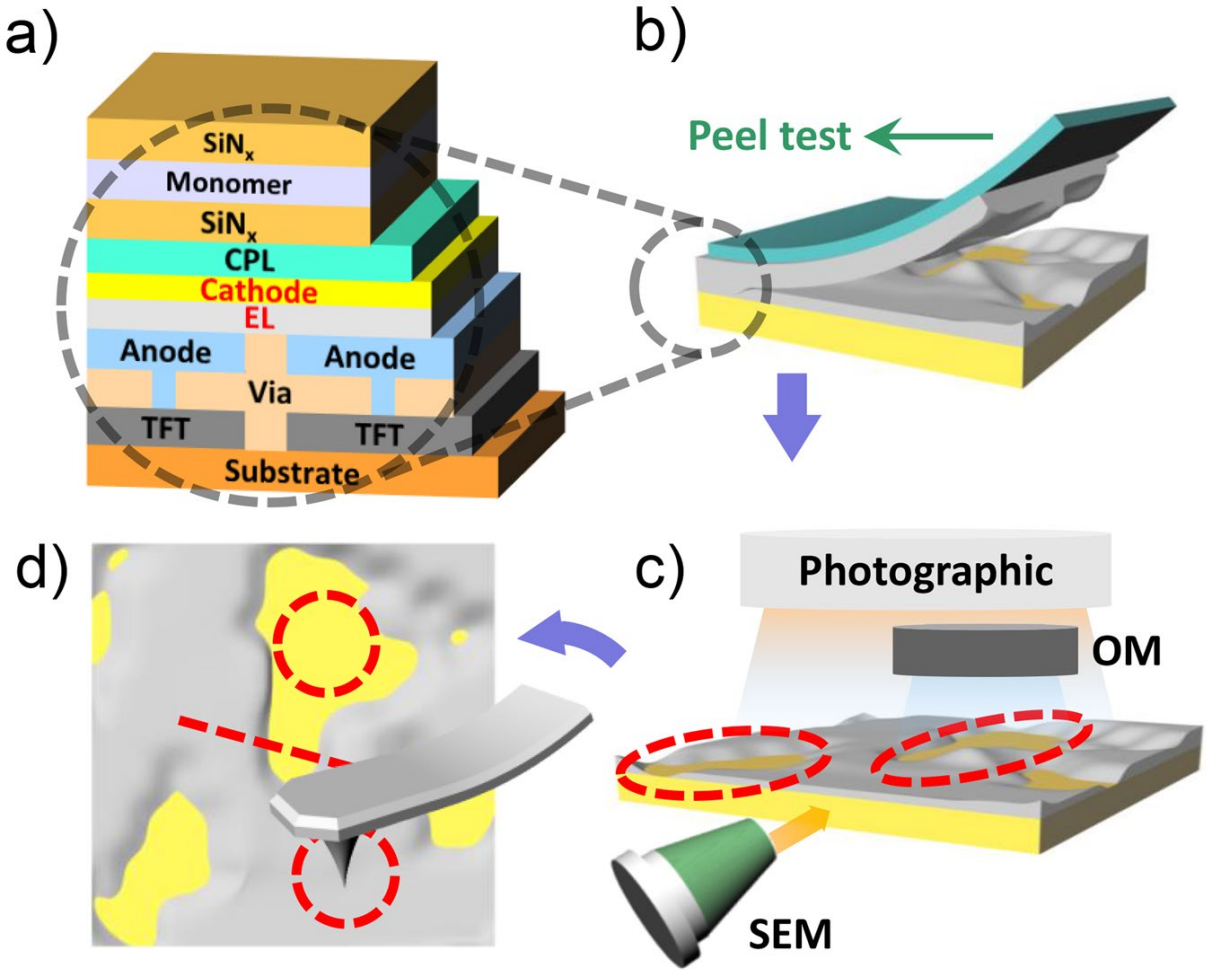
Schematics of (**a**) a representative structure of OLED and (**b**–**d**) experimental procedures for (**b**) peel tests, (**c**) photographic, OM, and SEM measurements on the exposed surface after the peel test, and (**d**) AFM measurements on the exposed surfaces.

Figure 1 shows schematics of the OLED structure and the experimental procedures. Generally, the OLEDs are multi-layered structures, consisting of an encapsulation layer (SiN_x_)/monomer/SiN_x_/capping layer(CPL)/cathode/EL/anode/Via/thin film transistor/substrate. In order to explore the adhesion properties of the OLEDs, the widely used peel test was employed as shown in Fig. 1(b)^1,13,24–26^. The peel test is a well-known technique for measuring macroscopically the adhesion properties of materials through the analysis of the maximum load because this maximum load is defined as the force measured when the layers completely detached from the fixed OLED. The details on the measurements can be found in methods section. In this way, the magnitude of the macroscopic adhesion force of each OLED was obtained. However, this method did not provide adhesion properties of each individual interface between layers, making it difficult to probe which layers demonstrated low or high adhesion. Thus, to explore the adhesion properties of the individual interfaces, we performed the following analysis procedure: (1) The peel test was performed to obtain the macroscopic adhesion force of the OLED (Fig. 1(b)). (2) Photographic and OM images were obtained to observe any exposed layers and their area portions over the OLED (Fig. 1(c)). (3) Cross-sectional SEM imaging was performed to identify each layer (Fig. 1(c)). (4) After specifying each layer, topography and adhesion force images were measured using contact and PinPoint^TM^ AFMs to probe surface roughness and the adhesion force, respectively, of each exposed layer, which are expected to be major factors contributing to macroscopic adhesion under fixed environmental conditions (Fig. 1(d)).

**Figure 2:**
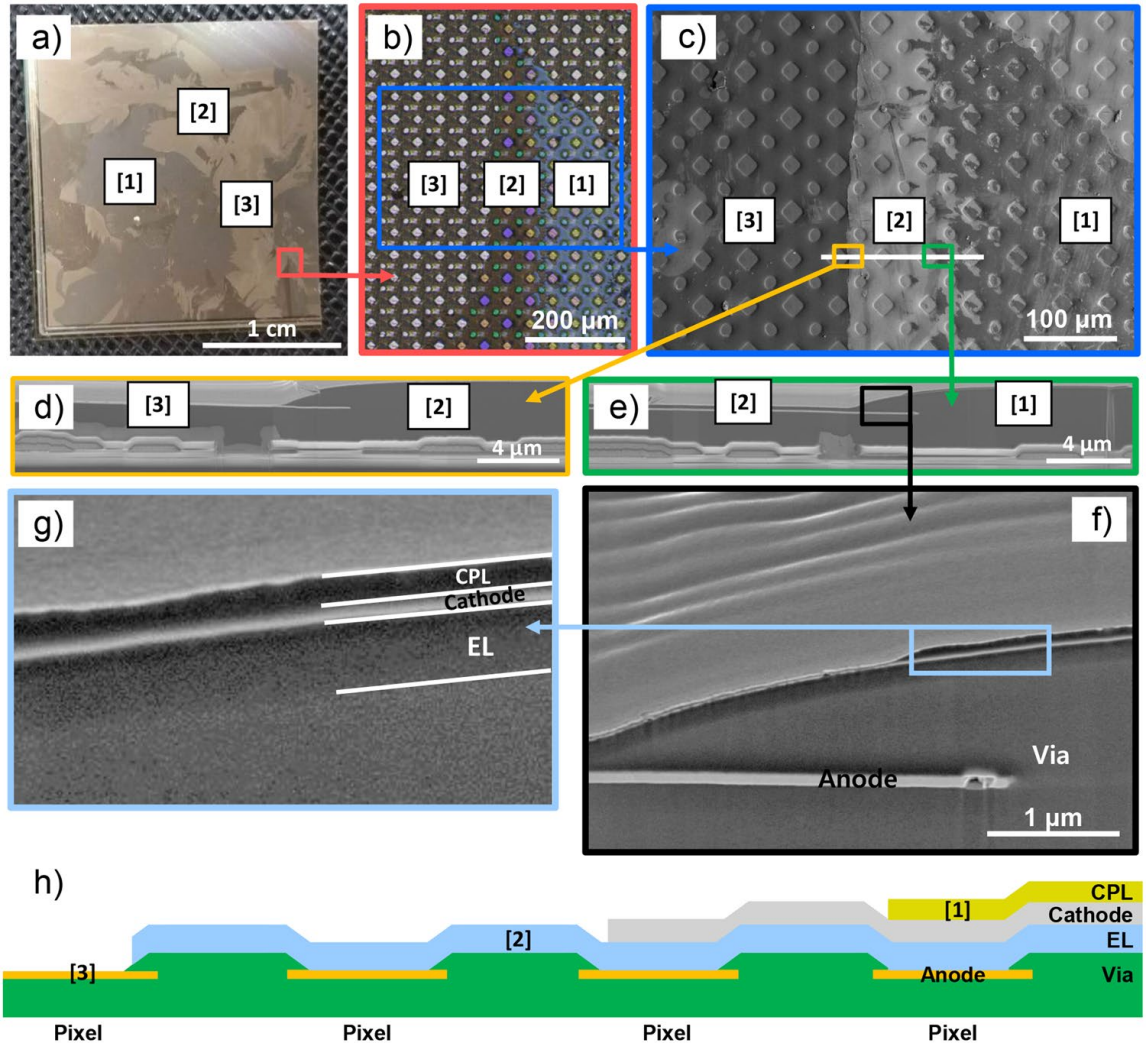
(**a**) Photographic image of an OLED after the peel test. (**b**) OM image magnified from red colored box in Fig. (**a**). (**c–g**) SEM images magnified from each colored region (see the arrows). (**h**)Schematic of OLED structure along the white line in Fig. (**c**).

As described above, to investigate the exposed layers, we acquired photographic and OM images by digital camera and OM, respectively, following the peel test. In the photographic image shown in Fig. 2(a), three layers were exposed after the peel test, a behavior that occurred for all of OLEDs (see Fig. S1). These three layers are better distinguished in the OM image of Fig. 2(b). We note that the circle and diamond like shape is a pixel, which is an illuminating part in OLED. The exposed layers are differentiated by the colors indicated such as blue, dark brown, and light brown, throughout the OM image. We designate the blue layer as Layer [1], the dark brown layer as Layer [2], and the light brown layer as Layer [3]. After the classification of each layer with the aid of the OM image, SEM measurements were carried out to further identify each exposed layer. We first acquired a plane-view SEM image as shown in Fig. 2(c), in which the region is also indicated as a blue box in Fig. 2(b). Similar to the OM image, the exposed three layers were clearly distinguished by contrast. To identify each layer in the OLED structure of Fig. 1(a), cross-sectional SEM images were acquired as shown in Fig. 2(d–g). Figures 2(d,e) show interfacial regions between the Layers [3] and [2] and between the Layers [2] and [1], respectively. This implies that the exposed layers are stacked in the order of Layers [1], [2], and [3]. To better observe interfacial regions, we observed the black box of Fig. 2(e) with a higher magnification as shown in Fig. 2(f,g). Considering the OLED structure in Fig. 1(a), these SEM images indicated that the anode was Layer [3], the cathode was Layer [2] and stacked on the EL/anode (Layer [3]), and the CPL was Layer [1] stacked thereupon. This also indicates that the interfaces between the anode and the EL, between the cathode and the CPL, and between the SiN_x_ and the CPL are weaker than those of the other interfaces. We note that there is a structural difference between the pixel part (diamond and circle like structures in OM and SEM images) and the non-pixel part. In particular, the anode and the EL layers are directly in contact with each other in the pixel part. Therefore, each exposed region can be represented as a schematic diagram shown in Fig. 2(h). We further note that the slightly darker contrast in the upper part of the EL layer (Fig. 2(g)) might be related to the artifact from the shadowing of the cathode layer because the cross-section was not perfectly smooth. In addition, we further confirmed each layer using X-ray photoelectron spectroscopy (XPS) (see Fig. S2).

**Figure 3:**
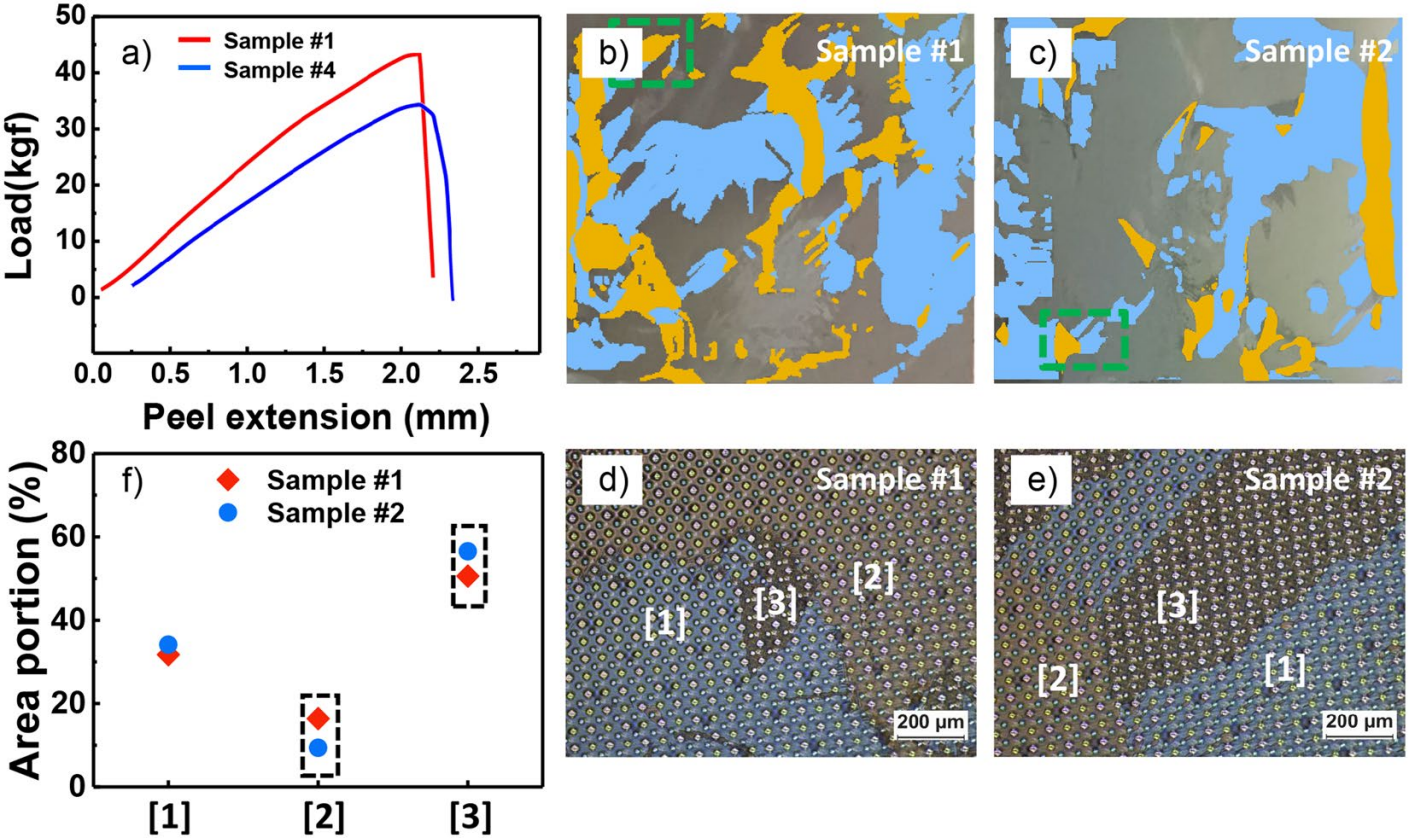
(**a**) A peel load-extension curve. (**b**,**c**) Photographic images obtained after the peel test for the exposed surfaces of the OLEDs (**b**) #1 and (**c**) #2, respectively. Note that the regions colored light blue and yellow refer to the Layers [1] and [2], respectively, and the rest of the regions refer to Layer [3]. (**d**,**e**) OM images of the dotted green boxes in Figs. (**b**,**c**) for the OLEDs (**d**) #1 and (**e**) #2, respectively. (**f**) Area portion as a function of the layers obtained from the image analysis.

After confirming the type of each layer from procedures (1) to (3), we chose two different OLEDs, of which show two extreme (smallest and largest) maximum loads in the peel test among 10 different OLEDs (see Table S1), and performed AFM measurements on these OLEDs with procedure (4). Figure 3(a) shows the peel load depending on the peel extension for two OLEDs. The maximum load of OLED #1 was higher than that of OLED #2, as shown in Fig. 3(a), meaning that the macroscopic adhesion force of OLED #1 over the measured region was relatively higher than that of OLED #2.

Even though peel load values are widely used to evaluate differences in macroscopic adhesion, such values cannot distinguish which interfaces are weaker than others or determine the contribution of a certain interface to the macroscopic adhesion. The weakest interface can be primarily responsible for the macroscopic adhesion measured by the peel test and, furthermore, can exhibit largest area portion among the exposed layers or interfaces. Thus, after identifying each exposed layer in a manner similar to Fig. 2, the area portion of each exposed layer was graphically calculated in each OLED using the photographic images. As mentioned above, all of the OLEDs showed the same exposed layers. The yellow and light blue regions in Fig. 3(b,c) indicate Layers [2] and [1], respectively, which were also confirmed from the OM images (Fig. 3(d,e)) taken at the dotted green boxes in Fig. 3(b,c). Figure 3(f) shows the area portion of each layer obtained from Fig. 3(b,c) by using the image analysis tool, ImageJ, showing that the area portion of Layer [1] was nearly the same in both OLEDs. This suggests that the area portion of Layer [1] had no significant effect on the peel test results. However, while the area portion of Layer [2] in OLED #1 was larger than that of OLED #2, the area portion of Layer [3] in OLED #1 was smaller than that of OLED #2. This implies that the difference in the ratio of the remaining portions of Layers [2] and [3] resulted in the difference of the maximum load in the peel test. Since the area portion of Layer [3] was much larger than that of Layer [2] for both OLEDs, the primary layer for determining the macroscopic adhesion is likely Layer [3], indicating that the interface between the anode (Layer [3]) and EL (Layer [2]) might significantly affect macroscopic adhesion. In other words, the relatively small area portion of Layer [3] in OLED #1 is related to its higher macroscopic adhesion.

**Figure 4:**
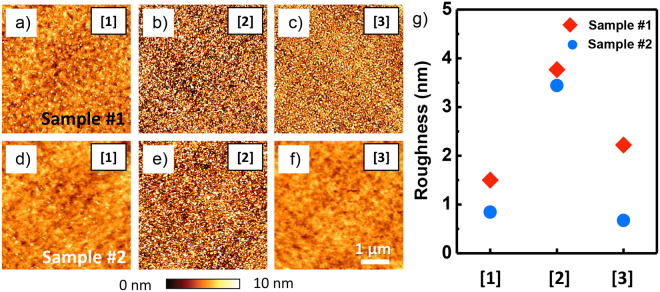
(**a–f**) Topography images measured on the Layers (**a**,**d**) [1], (**b**,**e**) [2], and (**c**,**f**) [3], respectively, for the OLEDs (**a–c**) #1 and (**d–f**) #2. The scale bar is 1 μm. (**g**) Roughness values of each layer for the two OLEDs.

After examining macroscopic approaches, a topographic imaging was performed on each layer, as presented in Fig. 4, visualize local differences in topographic features all the layers for both OLEDs appeared uniform over the measured area, but the detailed surface microstructures differed slightly. Layer [1] is a uniformly flat surface with a few particle-like features (see Fig. 4(a,d)). However, Layer [2] has a completely different topography, with a surface appearing to be very rough. Layers [1] and [2] show similar topography for both OLEDs. However, the topographic features of Layer [3] were completely different for OLEDs #1 and #2, with OLED #1 being rough with many particle-like features, and OLED #2 being rather flat. These topographic features can be confirmed by the roughness values shown in Fig. 4(g) that represent the root mean square (RMS) roughness for each topographic image. Note that, in general, macroscopic adhesion is correlated with the degree of surface roughness^27,28^. Therefore, if we consider a single OLED, Layer [2] may not contribute significantly to the observed macroscopic adhesion because its roughness is relatively higher than that of the other layers, and therefore, the weaker interfaces then eventually determine the observed macroscopic adhesion. Furthermore, Layer [2] might not contribute to the relatively different macroscopic adhesion observed in the present study, because the surface roughness for Layer [2] is similar for both OLEDs. Instead, since the difference in roughness between the OLEDs is the highest for Layer [3], the difference due to the Layer [3] may be responsible for the relative difference in macroscopic adhesion. The relative (absolute) roughness differences compared to the higher value between Layers [1] and [3] are 43.8% (0.658) and 69.7% (1.548), respectively. In other words, the interface between anode (Layer [3]) and EL might strongly affect to the macroscopic adhesion properties of the OLEDs.

**Figure 5:**
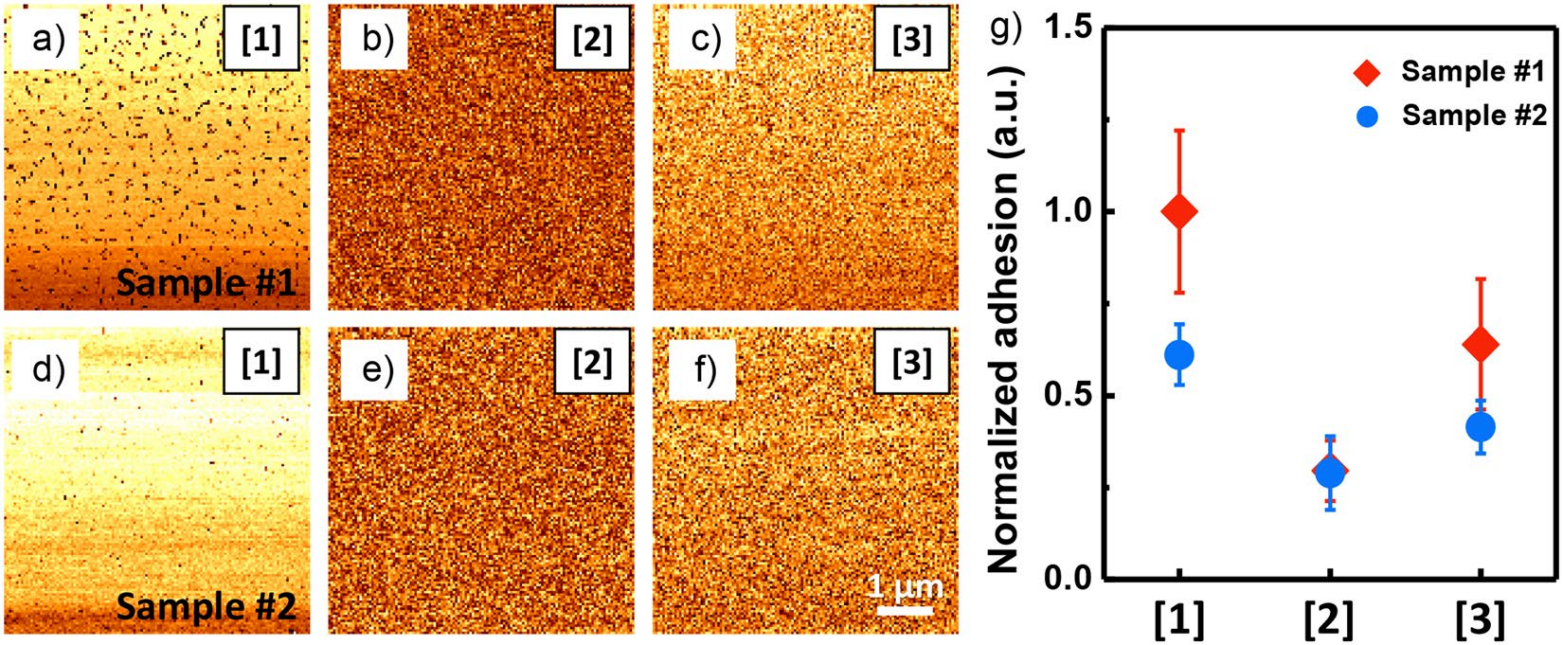
(**a–f**) Adhesion force images measured on the Layers (**a**,**d**) [1], (**b**,**e**) [2], and (**c**,**f**) [3], respectively, for OLEDs (**a–c**) #1 and (**d–f**) #2. The scale bar is 1 μm. (**g**) The normalized adhesion value for each layer for the two OLEDs.

In addition to the surface roughness, local adhesion can contribute to macroscopic adhesion. Thus, we measured the local adhesion of each layer using PinPoint^TM^ measurements with the AFM. Figure 5 shows the adhesion force images for each confirmed layer on both OLEDs. The normalized adhesion values are shown in Fig. 5(g). It presents that the average value and deviation of adhesion is normalized by maximum averaged value of the measured adhesion force among the layers. The different materials composing each layer can be clearly distinguished through the adhesion force images. Both OLEDs exhibited the same tendency, with Layer [1] having the highest adhesion force and Layers [3] and [2] larger yet, in order. Moreover, when comparing, the adhesion forces of Layer [2] in OLEDs #1 and #2 are nearly identical, but those for Layers [1] and [3] are different. Similar to the surface roughness which was discussed above and shown in Fig. 4, while Layer [2] demonstrates nearly the same adhesion, Layers [1] and [3] show a difference in adhesion, confirming that Layer [2] did not contribute to the macroscopic adhesion. The relative adhesion differences comparing the higher values between Layers [1] and [3] were 36.0% and 32.1%, respectively. Even though Layer [1] showed a slightly higher adhesion difference, the local adhesion in this study might not be a dominant factor in determining macroscopic adhesion because the relative adhesion difference for both OLEDs were small or negligible, considering the deviation and relative roughness difference were relatively large. This can be also observed from the macroscopic adhesion shown in Fig. 3. In addition, since the measured local adhesion is relative to the AFM tip, the actual adhesion may be slightly different. Nonetheless, if the relative adhesion difference is large, local adhesion may contribute to the macroscopic properties. Overall, the difference between the OLEDs of macroscopic adhesion as measured by the peel test might originate primarily from the roughness of Layer [3]. Thus, the macroscopic adhesion properties of the OLEDs were primarily influenced by the interface between the anode and the EL. This phenomenon may be occurred by the OLED preparation process. Up to the deposition process of the anode, the preparation of each layer was continuously done. However, after the deposition of the EL layer, there was some delayed time for the deposition of the EL layer because of the difference from inorganic and organic materials. Therefore, since the delayed time is longest among all other processes, the anode surface might be affected by environments, causing slightly different adhesion properties.

## Conclusion

In conclusion, we investigated the origin of local contributions to macroscopic adhesion in OLEDs using a combination of various microscopy techniques at different length scales. After performing a peel test, we identified two weak interfaces in the OLEDs that primarily contributed to macroscopic adhesion using a combination of photographic, OM, and SEM images. The three exposed layers remaining on the surfaces were the anode (Layer [3]), cathode (Layer [2]), and CPL (Layer [1]). Further, based on the analyses of the photographic images, the relative area portion between the exposed layers that interfaced between each anode (Layer [3]) and EL primarily affected macroscopic adhesion. Finally, after identifying each layer, the topography was characterized and the adhesion force images were acquired using AFM to probe local physical properties such as surface roughness and adhesion force. Even though no significant difference in local adhesion was found, visible differences in roughness were observed in Layer [3]. This confirmed that Layer [3], the interface between the anode and EL, was predominant in affecting the macroscopic adhesion properties of the OLEDs. As a result, by using a combination of various microscopy techniques at different length scales, weak interfaces in the OLEDs and the main contributors to macroscopic adhesion were revealed. These results could provide useful information on macroscopic adhesion of OLEDs as well as other devices composed of multiple layers. Furthermore, these observations provide guidelines for improving adhesion between interfacial layers in OLEDs.

## Methods

### OLED preparation and peel test

OLEDs were obtained from Samsung Display Co. Ltd. A subsequent peel test (INSTRON 5967) was performed on each OLED in order to peel the layers, demonstrating weak adhesion. The peel test is performed as follows: (1) Attaching each OLED to the Al plate by adhesive tape (to keep it from falling). (2) A mount jig was attached to the region where the peel test was to be performed (2 cm × 2 cm of surface area at the edge of OLED). (3) Fixing the Al plate to the peel test machine, the mount jig was lifted and the macroscopic adhesion was measured. The maximum load is defined as the force measured when the layers completely detached from the fixed OLED. The peel test was performed on 10 different OLEDs, and the other experiments were conducted on the largest and smallest of maximum load in OLEDs. The results measured by the peel test are attached in Table S1.

### Optical microscopy and SEM measurements

OM measurements were performed using a commercial instrument (DM2700, Leica). The OM images were acquired using the bright field mode at a magnification of 100× under ambient conditions. Plane-view and cross-sectional SEM images were obtained with an FEI Helios Nanolab 460F1 focused ion beam.

### Image analysis

Photographic images were taken with a common digital single lens reflex camera. Image analysis was performed using an open-source scientific image analysis tool ImageJ. Each layer in the photographic image was manually differentiated and the area portion was calculated.

### AFM measurements

A commercial AFM (NX-10, Park Systems) was used for adhesion force measurements. The PinPoint^TM^ measurement method was performed using a Si tip (CONTAl-G, BudgetSensors) for obtaining adhesion force images^29^. Typically, the local adhesion force is measured via a force-distance (F-D) curve^30^. In the F-D curve, the AFM tip initially contacts the sample surface, followed by separation. Here, the pull-off force is considered equivalent to the adhesion force. Note that the PinPoint^TM^ measurement technique acquires images for adhesion properties. It is considerably faster in acquiring the adhesion forces compared to the conventional F-D curve measurement techniques. During AFM measurements, the relative humidity and temperature were maintained at ~12% and 28.5 °C, respectively.

## Electronic supplementary material

SUPPLEMENTARY INFORMATION
