# Supplementary material for: Origin of macroscopic adhesion in organic light-emitting diodes analyzed at different length scales
Source: Sci Rep. 2018 Apr 23;8:6391. doi: 10.1038/s41598-018-24889-9 (PMC5913230; doi:10.1038/s41598-018-24889-9)
Supplement: Supplementary file 1 — SUPPLEMENTARY INFORMATION [file 41598_2018_24889_MOESM1_ESM.doc]

**Supplementary Information**

**Origin of macroscopic adhesion in organic light-emitting diodes analyzed at different length scales**

**Sungho Kim^1^, Seongjae Park^1^, Wanheui Lee^2^, Owoong Kwon^1^, Shang-U Kim^2^, Youngtae Choi^2^, Minyoung Yoon^1^, Jongwoo Park^2^, and Yunseok Kim^1,*^**

1 School of Advanced Materials Science and Engineering, Sungkyunkwan University (SKKU), Suwon, 16419, Republic of Korea.

2 Samsung Display Co., Ltd., Asan 31454, Republic of Korea.

*yunseokkim@skku.edu

**1. Peel test results**

**Table S1.** Peel test results of OLEDs.

| # | Maximum load (kgf) | # | Maximum load (kgf) |
| --- | --- | --- | --- |
| 1 | 43.499 | 6 | 39.954 |
| 2 | 34.903 | 7 | 37.709 |
| 3 | 42.924 | 8 | 35.628 |
| 4 | 39.201 | 9 | 35.026 |
| 5 | 37.928 | 10 | 35.505 |

We have measured 10 OLEDs. Among them, we chose two OLEDs (#1 and #2) which show two extreme (smallest and largest) maximum loads in the peel test.

**2. OM results**


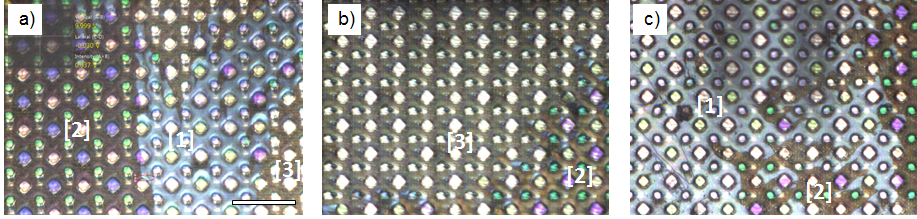


**Figure S1.** OM image in (a) #6 and (b, c) #8 OLEDs. Scale bar is 100 um.

In the OM images, all OLEDs show the same three explosed layers of which are Layers [1], [2] and [3], respectively, as presented in Fig. S1.

**3. XPS results**


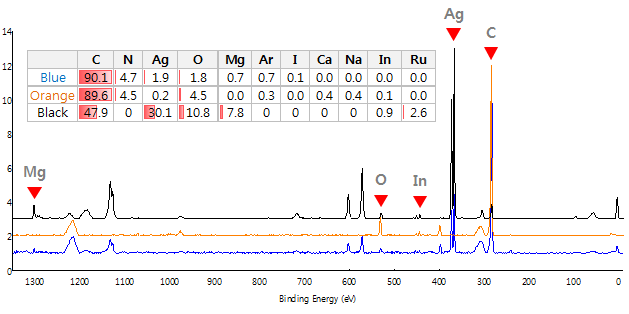


**Figure S2.** XPS results in OLED at various points.

To further identify each exposed layer, we performed X-ray photoelectron spectroscopy (XPS). Prior to the XPS measurements, surface ion etching has been performed by Ar ion over an area of 2 mm x 2 mm with 2keV for 90 second because of surface contamination of the OLED. Figure S2 shows XPS results at three different locations of each layer. Since the black colored line in Fig. 2 shows largest amount of Ag, which is included in the cathode, the black colored line corresponds to the results in Layer [1]. Similarly, since the blue colored line contains a small amount of Ag and largest amount of C, the blue colored line corresponds to the results in Layer [2]. We note that the small amount of Ag in the blue colored line can be detected in Layer [2] because the interface between the Layer [1] and [2] is not perfectly clear as shown in Fig. 2(c). As a result, the orange colored line corresponds to the results in Layer [3]. Indeed, each colored line was obtained in the Layers [1], [2], and [3], respectively.
